# A comparative analysis of human and AI performance in forensic estimation of physical attributes

**DOI:** 10.1038/s41598-023-31821-3

**Published:** 2023-03-23

**Authors:** Sarah Barrington, Hany Farid

**Affiliations:** 1 School of Information, University of California, Berkeley, USA; 2 Department of Electrical Engineering and Computer Sciences, University of California, Berkeley, USA

**Keywords:** Computer science, Computational science

## Abstract

Human errors in criminal investigations have previously led to devastating miscarriages of justice. For example, flaws in forensic identification based on physical or photographic evidence are notoriously unreliable. The criminal justice system has, therefore, started to turn to artificial intelligence (AI) to improve the reliability and fairness of forensic identification. So as not to repeat history, it is critical to evaluate the appropriateness of deploying these new AI forensic tools. We assess the feasibility of measuring basic physical attributes in a photo using a state-of-the-art AI system, and compare performance with human experts and non-experts. Our results raise concerns as to the use of current AI-based forensic identification.

## Introduction

Despite recent advances in artificial intelligence (AI) promising to revolutionise automated decision making, concerns are now being raised regarding fairness and efficacy across a range of high-impact fields, including the criminal justice system. The increasing use of algorithms in incarceration and rehabilitation has been widely scrutinized, ranging from policing^1^, to criminal sentencing^2^ and pretrial detention^3^.

 Use of these automated approaches has raised serious concerns regarding civil liberties and due process rights^4^. The COMPAS algorithm for predicting recidivism, for example, has been found to not only reinforce problematic racial and social biases^5^, but also perform no more accurately than untrained humans^6^. Similarly, in 2018, Buolamwini and Gebru found that popular facial verification and identification technologies— the use of which within law enforcement remains largely unregulated^7^—produced disproportionately higher error rates for racial minorities^8^.

It is, of course, appropriate to consider replacing or augmenting potentially error-prone human judgement and analysis with the goal of a more equitable criminal justice system. Here we focus on the growing trend of citizen policing in which, with a high-resolution camera in every hand, every-day citizens are playing an increasingly vital role in documenting everything from major global events to human-rights violations, police misconduct, and neighborhood crimes. At the same time, advances in artificial intelligence have made identifying individuals in images easier. And yet, reliable forensic identification is riddled with bias^9^ and errors^10,11^. The National Registry of Exonerations, for example, reports that between 1989 and 2019, flawed forensic techniques contributed to almost one quarter of wrongful convictions in the US. Some effort has gone into documenting and trying to address these issues in AI-based face recognition^12^, but less attention has been paid to basic forensic identification based on physical traits like height and weight.

To illustrate this point, in 2008 George Powell was identified as a suspect in a string of armed robberies. A store clerk initially identified the robber as 5$^\prime$6$^{\prime\prime}$ tall, and eventually identified Powell in a lineup. Powell stands at 6$^\prime$3$^{\prime\prime}$. From video surveillance, an expert measured the robber to be 6$^\prime$1$^{\prime\prime}$. Powell was convicted and sentenced to 28 years in prison. After his conviction, two new experts concluded the robber was less than 5$^\prime$10$^{\prime\prime}$, after which the original expert adjusted his estimate to a range of 6$^\prime$1$^{\prime\prime}$ to 5$^\prime$10$^{\prime\prime}$. Due in part to these inconsistencies, Powell’s conviction was vacated in 2018, and he was granted a new trial.

Because physical attributes like height, weight, age, and race are fundamental to forensic identification, it is essential to validate the accuracy of new and traditional tools. Height and weight estimation could also play a crucial role in increasing the reliability of photographic identification. If, for example, weight can be estimated to within an accuracy of $5\%$, then based on the distribution of US adult male weights^13^, some $90\%$ of men could be eliminated from consideration from this single measurement.

Despite its seeming simplicity, many factors make it challenging to accurately estimate height and weight from a single image. Due to spinal compression, for example, height fluctuates daily by up to 1.9 cm^14^; due to body pose, apparent height in an image can vary by up to 6 cm^15^; and shoes, hair, and headwear further obscure a person’s true height.

Recent advances in AI and computer vision have led to spectacular leaps in image understanding and modeling of the human form (e.g.,^16,17^). We evaluate the accuracy with which AI-based tools—and for comparison—expert photogrammetrists and non-experts can estimate a person’s height and weight from a single image.

## Materials and methods

### Data set

**Figure 1:**
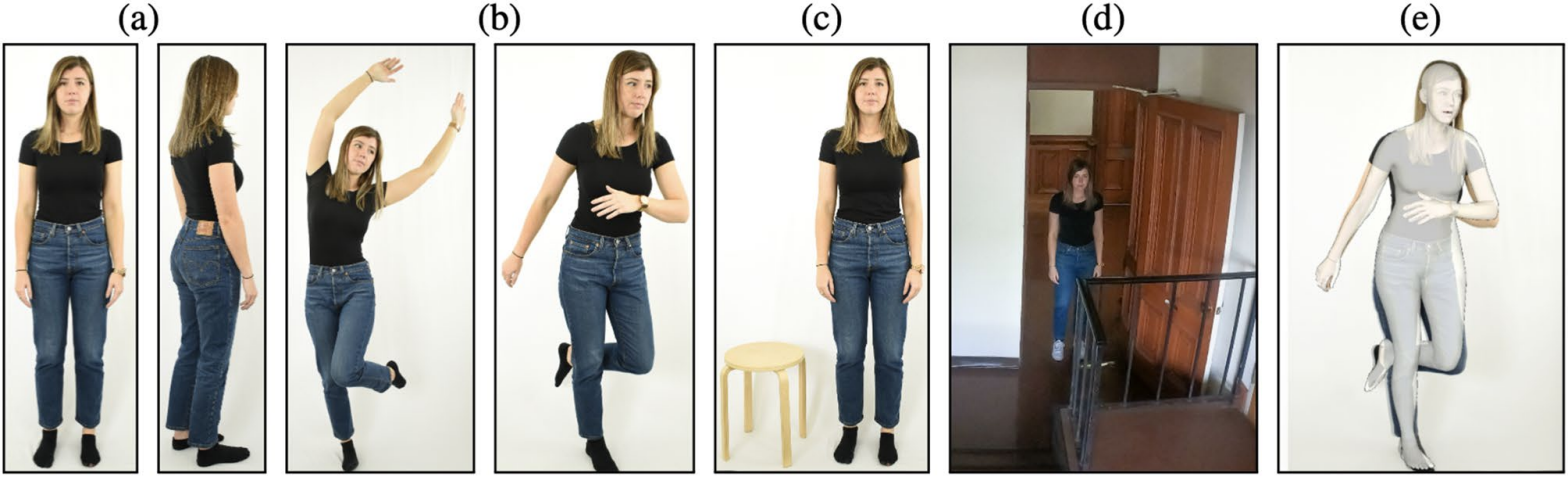
Representative examples (published with permission of the participant) of the calibrated data set consisting of (**a**) no-reference studio (neutral poses); (**b**) no-reference studio (non-neutral poses); (**c**) reference studio; and (**d**) in-the-wild. Shown in panel (**e**) is a representative example of 3D model fitting to the image in panel (**b**).

A total of 58 participants (33 women and 25 men) were recruited from the UC Berkeley campus and photographed in two settings: (1) a studio setting with a fixed white background and artificial lighting with a tripod-mounted DSLR camera (4000 $\times$ 6000 pixels); and (2) an in-the-wild setting emulating a CCTV-like scene in which a narrow corridor was photographed by a ceiling-mounted GoPro camera (5184 $\times$ 3888 pixels). Each participant was assigned an anonymized identifier and photographed in the studio setting in eight neutral poses, Fig. 1a, six dynamic poses, Fig. 1b, and one neutral pose while standing next to a reference object (the same stool was used for all participants), Fig. 1c. Each participant was photographed in the wild in two static, Fig. 1d, and three dynamic poses. This process yielded a total of 812 no-reference studio images, 58 reference studio images, and 290 in-the-wild images.

Each participant’s height and weight was measured and recorded alongside their anonymized identifier. The collected female/male heights are normally distributed with a mean of 161.1/176.1 cm and a standard deviation of 5.3/8.3 cm; the average US adult female/male height is 161/175 cm with a standard deviation of 7.0/7.4 cm^13^. The collected female/male weights are 60.9/78.4 kg with a standard deviation of 11.4/12.9 kg; the average US adult female/male weight is 78.7/90.8 kg with a standard deviation of 19.7/19.8 kg^13^. While our participants’ heights closely follows the national average, our participants weighed approximately $20\%$ less than the national average and are less variable (presumably because they were drawn primarily from a University student population). Each participant was paid $\$20$.

### AI

Recent advances in machine learning and computer vision have led to impressive results for estimating body shape and pose from a single image^16^. We previously extended this system to yield state-of-the-art body shape and pose estimation^18,19^. Here we briefly describe this system.

A full-body, 3D model is fit to an image of a person using an augmented version of SMPLify-X^16^. The original SMPLify-X extracts 2D keypoints from the body and face, from which a 3D model is automatically fit. Although this model can accurately capture complex body poses, it does not incorporate body shape. This is because the model fitting relies only on the extracted 2D skeletal keypoints and does not consider the body shape depicted in the image. An augmented version^19^ of this model incorporates into the 3D modeling an additional parameter that captures the overall body shape, yielding more accurate estimates of body shape and size, Fig. 1e.

Although the 3D body model is estimated in real-world units, this metric reconstruction is highly inaccurate^18^, even while the overall body pose and shape are well estimated. We, therefore, adopt a different approach that scales the estimated 3D model based on a gender-specific average inter-pupillary distance (IPD). The IPD is relatively consistent, with an average adult IPD for women/men of 6.17/6.40 cm with a standard deviation of 0.36/0.34 cm^20^. Because our 3D models do not have pupils, the pupil center is specified as the midway point between the left and right corners of the eye.

Once scaled, the 3D model is reposed into a neutral, upright pose, from which the person’s height is measured as the distance from the top of the head to a plane formed by three points on the bottom of the feet. The person’s weight is measured as the volume of the 3D model, converted to kilograms by multiplying by 1023 kg/m$^3$, corresponding to a gender-agnostic average body fat of $34\%$^21^.

### Experts

We recruited 10, US-based, certified photogrammetrists (certification requires a minimum of between four and six years of experience depending on the governing body). Each expert was provided with a random subset of five in-the-wild images (each image depicted a different person) and asked to estimate the person’s height and weight (one expert declined to estimate weight). Each expert was provided with a schematic diagram of the scene with two real-world measurements consisting of the width of the back door into the hallway and the distance between the back door and the top of the stairs.

### Non-experts

We recruited 325 participants from Amazon’s Mechanical Turk platform. Unlike the experts described in the previous section, who made height and weight estimates from only the in-the-wild images, our non-experts were tasked with making estimates from the no-reference studio images, the referenced studio-images, or the in-the-wild images. A representative subset of 290 (out of 812) no-reference studio images were partitioned into five non-overlapping sets of 58 images in which each photographed participant appeared only once. The 290 in-the-wild images were similarly partitioned into five non-overlapping sets of 58 images each. The 58 reference studio images were placed into a single set.

On entry into the study, each participant was assigned a random set from the above 11 possible subsets. Shown one image at a time, in random order, participants were asked to estimate the height and weight of the person depicted in the photo. Unlike the experts and AI, no additional information was provided to these non-experts.

Randomly interspersed within the 58 images were four catch trials consisting of stock photos clearly annotated with the subject’s height and weight. If a participant failed any of the catch trials, their entire set of responses were excluded. A total of 65 out of 325 participants failed to correctly complete the catch trials, and another 24 failed to complete the study, yielding a total of 236 valid responses. Participants were paid $5.00, but were not paid if they failed any of the catch trials. Each image was analyzed by an average of 22 non-experts.

Denoting the estimated height from non-expert *j* for image *i* as $\tilde{h}_{i,j}$ with true height $h_{i}$, the median *individual* accuracy is computed as $\text{ median}_j\left( |\tilde{h}_{i,j} - h_{i} |\right)$; the median *crowd* accuracy is computed as $|\text{ median}_j(\tilde{h}_{i,j}) - h_{i} |$. The individual and crowd weight errors are estimated in the same way. The median error across all images are reported in Table 1 in both absolute units (cm/kg) and as a percent of base height and weight. A median (as compared to a mean) is employed because responses within and across images are not normally distributed.

### Human subjects

All data collection was approved by the UC Berkeley Committee for Protection of Human Subjects (2022-01-14999). All participants provided informed consent prior to their participation, and data collection was performed in accordance with relevant guidelines and regulations.

**Figure 2:**
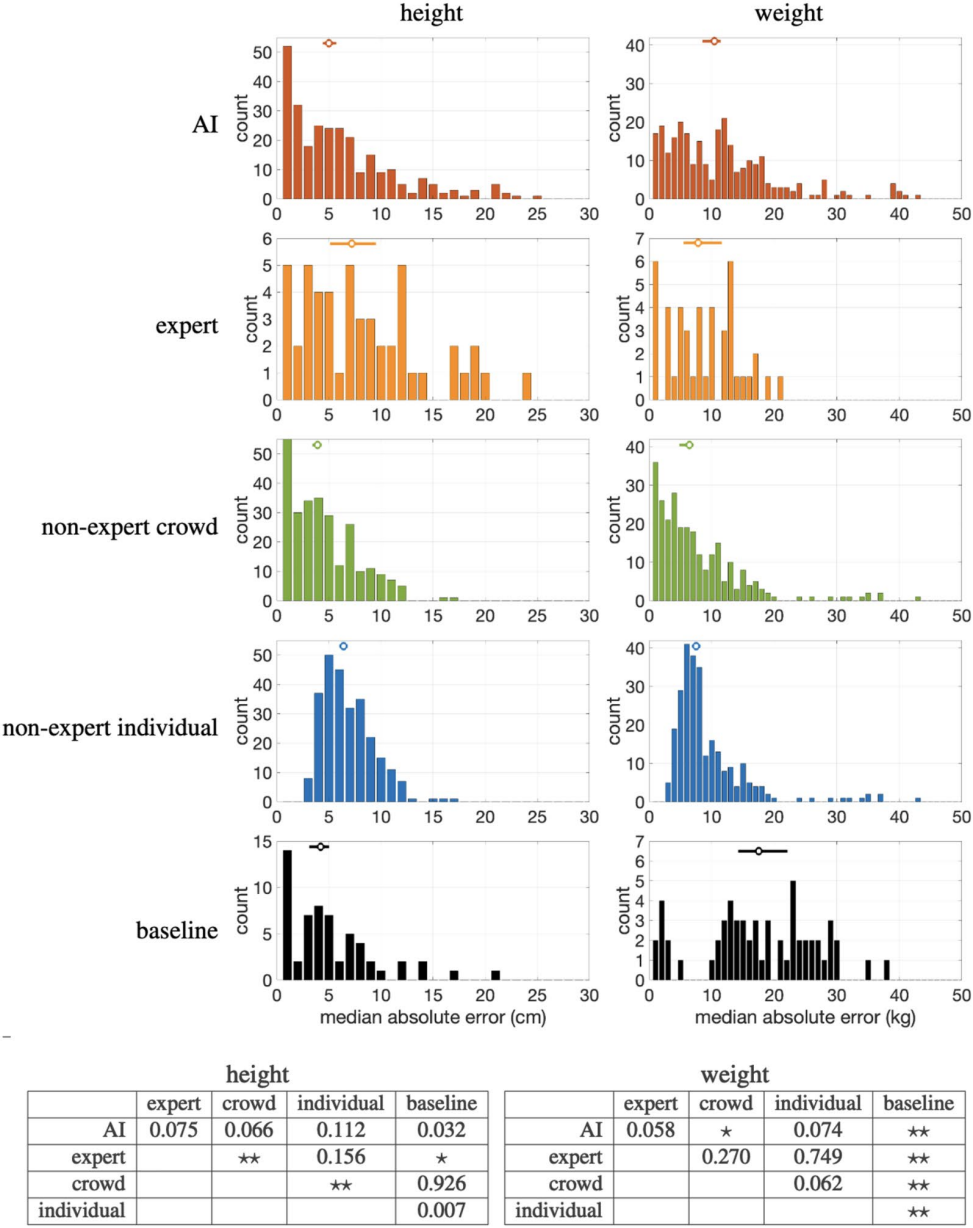
The distribution of in-the-wild height/weight errors for AI, expert, non-expert crowd, non-expert individuals, and baseline. The open circles and horizontal error bars correspond to the median error and $95\%$ confidence intervals. The two tables denote the pair-wise statistical significance at $p<0.005$ ($\star$) or $p<0.0005$ ($\star \star$) between different groups. See also Table 1.

## Results

Shown in Table 1 is a summary of the height/weight estimation errors for AI, expert, non-expert, and baseline from 1160 images across our three data sets (Fig. 1). Shown in Fig. 2 are the error distributions annotated with the median and $95\%$ confidence intervals computed from 1000 bootstrap iterations.

The baseline estimator corresponds to simply using a gender-specific average US adult height/weight for every image (see “Data set” in “Materials and methods”). With a median height error of only 4.2 cm, this baseline predictor is surprisingly good, outperformed only by the non-expert crowd. With a median weight error of 17.5 kg, however, the baseline is the worst performing. This asymmetry is due to the fact that gendered adult heights have relatively low variance as compared to weight.

At first glance, the non-expert crowd is more accurate than all others even in the no-reference studio images in which height/weight estimates are made in the absence of any contextual information (Fig. 1a, b).

Of the 290 in-the-wild images, we obtained height/weight estimates from all groups for 50/44 images (one expert declined to estimate weight). From this subset, a 5-way Friedman test reveals a significant difference in the error distribution of height ($p = 3.5\times 10^{-6}$) and weight ($p = 9.8 \times 10^{-6}$). Following this, we performed 10 Wilcoxon two-sided rank tests on all pairs of height/weight estimates. Shown in the lower portion of Fig. 2 are the resulting *p*-values where statistical significance is set at $p < 0.005$, incorporating a Bonferroni correction to adjust the baseline p-value of 0.05 by the 10 pairwise comparisons.

The AI-based height estimator is no more accurate than experts, non-experts, or baseline (guessing a gender-specific average height). Experts are no more accurate than individual non-experts, and are less accurate than the non-expert crowd and baseline. Neither the non-expert crowd nor individual are more accurate than baseline.

The AI-based weight estimator is no more accurate than experts and individual non-experts and is less accurate then the non-expert crowd; and experts are no more accurate than non-experts. Unlike height, baseline weight is less accurate than all other groups. This asymmetry is due to the fact that the variance in adult weight is much higher than in height.

What is particularly surprising about these results is that both the AI and experts had access to explicit metric measurements (IPD and door/hallway measurements, respectively), whereas the non-experts were not provided this information.

It can be argued that these results only hold for our particular AI-based estimator. However, other state of the art AI estimators are as, or less, accurate than ours^23^. We contend, therefore, that the problem of accurate height and weight estimation may be out of reach of current AI systems.

**Table 1:**
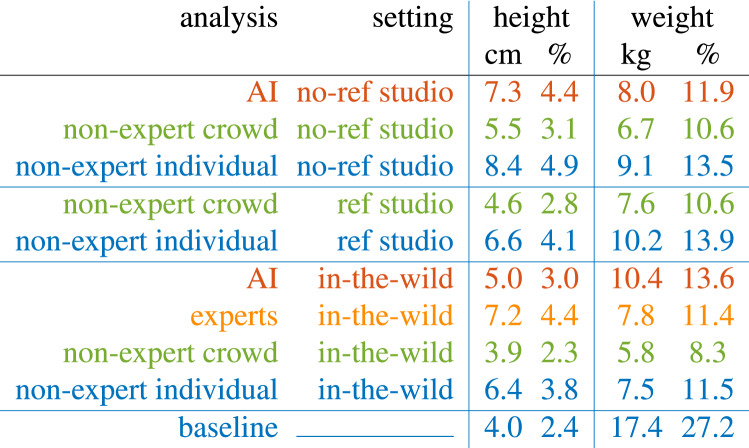
Median height/weight error in absolute units (cm/kg) and as a percentage (%) of base height/weight. See also Figure 2.

## Discussion

A group of two dozen non-experts outperforms AI and expert height/weight estimation even when the non-experts are provided with less information. This underwhelming performance by experts and AI should give significant pause as to how—or even if—it is reasonable to rely on these methods for forensic identification based on basic physical attributes. With a median AI-based height error of $4.4\%$, for example, a man standing at 183 cm ($6^\prime$) will be estimated to within a range of 175–191 cm ($5^\prime 9^{\prime\prime}-6^\prime 3^{\prime\prime}$), capturing a quarter of all US adult men.

Our experiments were not designed to evaluate gender or racial bias, however, we qualitatively find that height and weight errors are similar for women and men; we did not have enough diversity in our data set to determine if there are any racial biases. As with any forensic identification, it will be important to determine if any such racial (or other) bias exists.

The troubling state of human-based forensic identification needs critical attention^10,11^. Simply deploying AI-based tools, however, provides no guarantee that critical decision-making in criminal investigations will be any more fair or accurate, and—as our results reveal—they may make things worse. As with other automated techniques designed to replace or augment human decision making, it is critical to carefully evaluate the accuracy and potential bias in any such proposed systems. Most AI and computer-vision systems, however, are typically evaluated against previously published systems and are not directly compared to human performance. As it pertains to the criminal justice system, a machine-to-human comparison is critical to ensure that replacing or augmenting humans will not, in fact, lead to worse outcomes.

One advantage of the AI-based system evaluated here is that it explicitly estimates a person’s body shape and pose, from which height and weight can be explainably determined. By contrast, purely machine-learning based approaches take a more opaque approach, attempting to learn the relationship between an image of a person and their physical attributes. In the work of^24^, for example, the neural-network based system achieves a mean absolute height error of 8.4 cm for neutral poses and 12.1 cm for non-neutral poses; significantly worse than those reported in Table 1. In addition to the poor performance, this approach is not particularly explainable which—we contend—can be problematic in the criminal justice system where experts, attorneys, and judges should be able to scrutinize the inner workings of any forensic technique being used in such a potentially high-stakes setting.

We have focused on forensic identification based on height and weight. Even this most basic of measurements appears to be out of reach of modern AI-based systems, casting significant doubt as to the feasibility of AI-based forensic identification based on more complex measurements or features.

## Data Availability

Ground-truth height/weight measurements and AI, expert, and non-expert estimates are available at^22^.
